# Gonorrheal Infection

**Published:** 1894-08

**Authors:** 


GONORRHEAL INFECTION.—It has been shown by Brewer that in a case of gonorrheal infection, six years had elapsed during which no secretion could be pressed from the urethra, yet the microscope showed several characteristic colonies of gonococci. Six weeks afterward, contrary to advice, this man married and in two weeks communicated the infection to his wife in whom it went through the various stages of inflammation and resulted in abscess.—*Ex.*

